# Genomic studies of the additive and dominant genetic control on production traits of *Euterpe edulis* fruits

**DOI:** 10.1038/s41598-023-36970-z

**Published:** 2023-06-16

**Authors:** Guilherme Bravim Canal, Gabriela França Oliveira, Francine Alves Nogueira de Almeida, Marcello Zatta Péres, Gabriel Lenen Javarini Moro, Wagner Bastos dos Santos Oliveira, Camila Ferreira Azevedo, Moysés Nascimento, Marcia Flores da Silva Ferreira, Adésio Ferreira

**Affiliations:** 1 Department of Agronomy, Federal University of Espírito Santo, Alegre, Espírito Santo 29500-000 Brazil; 2 Department of Statistics, Federal University of Viçosa, Viçosa, Minas Gerais Brazil

**Keywords:** Agricultural genetics, Genetic markers, Genomics, Plant breeding, Plant genetics, Quantitative trait, Genetics, Plant sciences

## Abstract

In forest genetic improvement programs for non-domesticated species, limited knowledge of kinship can compromise or make the estimation of variance components and genetic parameters of traits of interest unfeasible. We used mixed models and genomics (in the latter, considering additive and non-additive effects) to evaluate the genetic architecture of 12 traits in juçaizeiro for fruit production. A population of 275 genotypes without genetic relationship knowledge was phenotyped over three years and genotyped by whole genome SNP markers. We have verified superiority in the quality of the fits, the prediction accuracy for unbalanced data, and the possibility of unfolding the genetic effects into their additive and non-additive terms in the genomic models. Estimates of the variance components and genetic parameters obtained by the additive models may be overestimated since, when considering the dominance effect in the model, there are substantial reductions in them. The number of bunches, fresh fruit mass of bunch, rachis length, fresh mass of 25 fruits, and amount of pulp were strongly influenced by the dominance effect, showing that genomic models with such effect should be considered for these traits, which may result in selective improvements by being able to return more accurate genomic breeding values. The present study reveals the additive and non-additive genetic control of the evaluated traits and highlights the importance of genomic information-based approaches for populations without knowledge of kinship and experimental design. Our findings underscore the critical role of genomic data in elucidating the genetic control architecture of quantitative traits, thereby providing crucial insights for driving species' genetic improvement.

## Introduction

*Euterpe edulis,* commonly known in commercial settings as "juçaizeiro," is a medium to tall palm tree that easily exceeds ten meters in height^1,2^. The species has shown great potential for use in fruit growing due to its excellent quality fruit and pulp with beneficial health attributes. In addition, this species has high fruit production and represents sustainable crop diversification with the possibility of management in native areas, as well as significant socioeconomic and ecological potential impacts. Another important factor highlighting the juçaizeiro in the fruit industry is the potential to sustain the market chain of açai pulp and sorbet^3^. Açaí is a product derived from the processing of fruits from species of the genus *Euterpe*^4^, and on a production scale, the raw material for this product comes almost exclusively from *Euterpe oleracea* (açaí palm). Although the processed products from both species are similar, the pulp of the juçaizeiro shows superiority in terms of nutritional, mineralogical, energetic, and flavor aspects^5,6^.

The açaí palm, in turn, requires higher temperatures and high water availability, limiting the geographic region for cultivation^7^. The juçaizeiro, naturally occurring throughout the Atlantic Forest^8–10^, shows a high level of phenotypic plasticity due to the great environmental variation resulting from the latitudinal range of this biome. Therefore, the juçaizeiro has the potential for cultivation in climatic zones not recommended for the açaí palm^7^.

Despite all these advantages, the species does not have registered varieties available in the seed market, which generates concerns regarding its acceptance in establishing commercial orchards. To that end, genetic improvement programs play a fundamental role in acceptance of the culture. They aim to promote procedures that provide more productive and higher-quality industrial varieties. However, due to the long juvenile period of the species, improvement cycles are long and costly and may require more than 15 years between recombination and the development of a new variety.

The non-domesticated status of the juçaizeiro means that the current technique applied to the culture is phenotypic selection, with a focus on additive terms, given that the species is exclusively propagated by seeds. However, a deeper understanding of the genetic architecture of agronomic traits can help breeders maximize genetic gains in each selective cycle^11^, and obtaining precise estimates is of great importance since genetic parameters such as heritability are among the most essential for determining the potential for genetic progress of the program^12^.

To estimate variance components and genetic values of individuals, it is common to use the information on genetic kinship through genealogy, which is represented by the A matrix, using the restricted maximum likelihood (REML) and the best linear unbiased predictor (BLUP) methods^13^. However, although effective, the results may be inflated since the genetic information matrix assumes unrealistic conditions, such that individuals from the same family share the same degree of genetic similarity^14^. As a result, variance components are overestimated, leading to inflated predicted genetic values^15,16^. This effect is amplified for analysis of non-domesticated species evaluated in natural populations without prior knowledge of parents, making genealogical information among individuals nonexistent, as is the case for juçaizeiro. This lack of information can result in overestimating additive genetic variance components and inflating genetic values^15^.

However, the increased accessibility of high-density genome sequencing techniques^15,17^ has enabled the acquisition of genomic information from individuals of interest, allowing breeders to construct the realized relationship matrix (G matrix), which represents the genetic sharing among individuals, and can replace the pedigree-based relationship matrix (A matrix)^18^. Substitution of the A matrix for the G matrix results in the genomic best linear unbiased predictor (GBLUP) method^19^, which can provide more accurate predictions of individual genetic values^14,17^. The use of genomic markers represents an enormous opportunity for non-domesticated species, as it allows decomposition of the genetic variance into its additive and non-additive terms, which would not be possible under normal conditions with unknown parents, providing fundamental information to breeders on the genetic control of the traits studied. The Diversity Array Technology Sequencing (DArTseq) approach uses high-density molecular markers for genomic analysis with broad genomic coverage, efficiency in detecting genetic polymorphisms, ease of analysis, and low cost^20–22^.

Although non-additive effects, such as dominance, are not currently considered in selective practices for juçara palm due to its exclusively sexual propagation via seeds, studies on forest tree species have demonstrated that incorporating dominance effects into statistical models improves the accuracy of heritability, predictive abilities, and influences on genetic gains^23–26^. In addition, the use of molecular markers can represent significant savings for breeding programs, in terms of time and cost, since establishing and developing experimental plots, as well as conducting controlled crosses in species for which there is limited knowledge of their pollination system, can be complex and require greater financial resources to develop a complete pedigree for in-depth evaluation of the genetic control of traits of interest. Thus, genetic markers can be a viable alternative to accelerate the more comprehensive evaluation of genetic control of traits and provide more information to breeders.

Given that stated above, this study aimed to use a whole genomic genotyping of DArTseq-derived SNPs to provide insights into the genetic structure controlling traits of interest to improve fruit production of the juçara palm tree, using BLUP and GBLUP approaches, as well as comparing the efficiency of models with different genetic effect structures.

## Material and methods

### Plant material and conducting experiments

**Figure 1:**
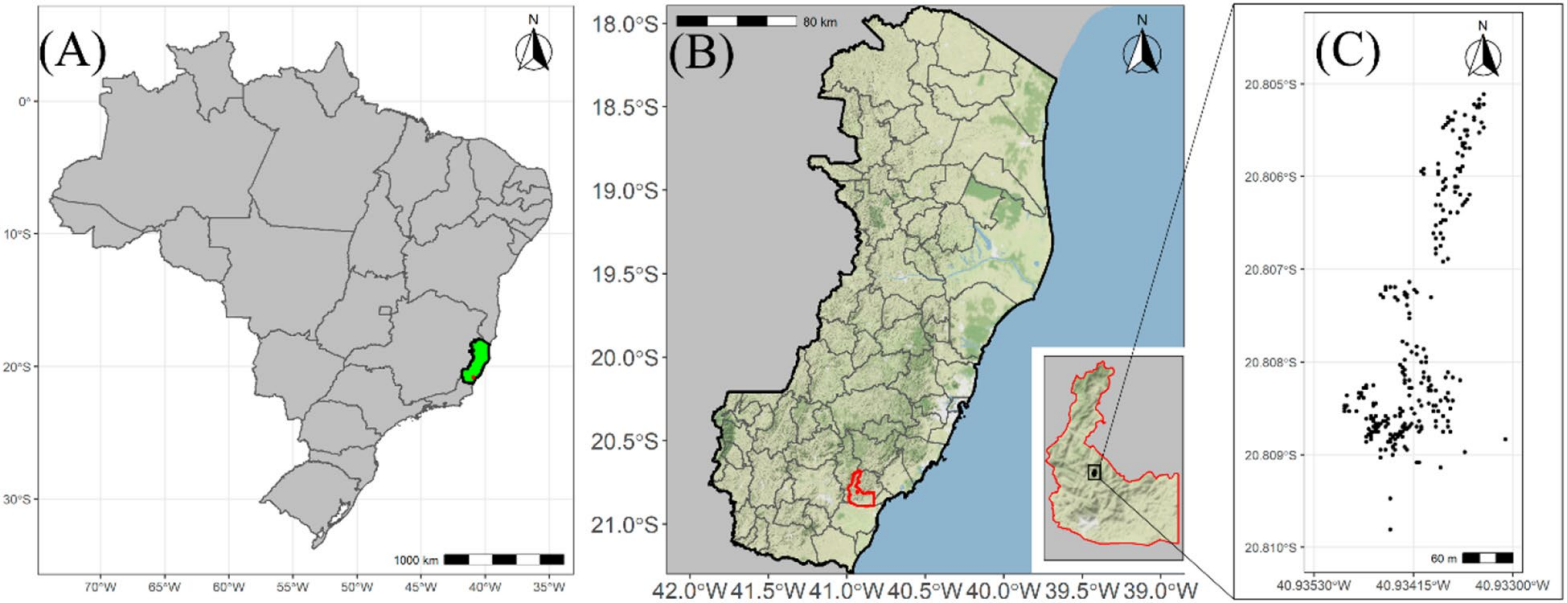
(**A**) Geographic representation of Brazil, with the state of Espírito Santo identified in green and a red dot denoting the geographical position of the experimental field; Map generated with R free environment *software.* (**B**) Espírito Santo, focusing on the municipality of Rio Novo do Sul and the location of the experimental area; (**C**) Geographical distribution of the genotypes evaluated in the orchard of the experimental area, represented by black dots. Map generated with R version 4.2.0^27^ free environment software, using the ggmap^28^, geobr^29^ and ggplot2^30^packages. The vegetation cover layer was sourced from the open-source Stamen Map server.

The study population consisted of a commercially managed juçaizeiro (*Euterpe edulis*) plantation, resulting from the process of natural recomposition and seed dispersal in a pasture area located in the municipality of Rio Novo do Sul, state of Espírito Santo, Brazil (Fig. 1). Consequently, field evaluations were conducted in the absence of an experimental design, with phenotyping carried out from 2018 to 2021. In 2020, only the number of bunches per plant (NBP) was counted.

Due to variations in fruit production and the restrictive measures associated with the COVID-19 pandemic, the years of evaluations and the number of genotypes evaluated for each trait differed. The initial determination of plants to be evaluated in the research followed two basic criteria: they had to have reached physiological maturity, presenting full fruit production, and they had to be in good phytosanitary condition, free of pests and diseases.

### Phenotyping

In this study, 12 productive and industrial traits of interest were evaluated, namely:

*Number of bunches per plant (NBP)* measured throughout the four years of the study, evaluated by counting the number of bunches with fruit.

The other variables were evaluated in 2018, 2019, and 2021. Fruits were harvested only when they reached the point of industrial maturation, which was determined every year by the same employee at the juçaizeiro fruit processing plant. After extraction of the fruit bunch, the following traits were evaluated:

*Fruit weight per bunch (kg) (FWB)* by weighing in kilograms (kg) the fruits extracted from the bunch of each genotype.

After extracting the fruit, the number of rachillas per bunch (NRB) was measured in these genetic materials by counting the number of rachillas inserted into the center rachis, which was then measured using a tape measure, evaluating the rachis length (cm) (RL) in centimeters (cm).

A fruit sample of each genotype was obtained, packed in properly identified plastic bags, and sent to the Plant Biometry Laboratory at the Federal University of Espírito Santo, where morphometric evaluations of fruit and seeds were carried out following a completely randomized design.

The weight of 25 fruits (g) (WF) and the weight of 25 seeds (g) (WS) were evaluated in grams (g) in four replications per genotype. With this information, the difference between the morphometric traits of fruits (WF) and the morphometric traits of seeds (WS) resulted in the amount of pulp in 25 fruits (g) (AP) in grams. The following formula was used to obtain the pulp yield (%) (PY):

$$PY=\frac{AP}{WF}*100$$

where *AP* is the amount of pulp and *WF* is the fresh mass of 25 fruits.

To evaluate the equatorial and longitudinal diameters of fruits and seeds (EDF, LDF, EDS and LDS, respectively) (mm), five fruits were measured in millimeters (mm) using a digital caliper. Marçal et al. (2016)^31^ concluded that five measurements are necessary to achieve a 95% coefficient of determination — although they provide additional information on the studies, measurements beyond this number increase cost and evaluation time.

### Genotyping and quality control of molecular markers

Genomic DNA was obtained from leaf samples of the 275 genotypes following the CTAB method^32^ with modifications^33^. After verifying the quantity and quality using a NanodropTM 2000 spectrophotometer (Thermo Scientific), the genomic DNA was sent to Mexico for genotyping high throughput using the DArTseqTM methodology at the Genetic Analysis Service for Agriculture (SAGA).

The sequences were analyzed using Dartsoft14, an automated analysis program of genomics data, and DArTdb, a laboratory management system, developed and patented by DArT Pvt. Ltd. (Australia), generating SNP marker data as described by Killian et al. (2012)^34^ and Sansaloni et al. (2020)^22^. Additional information can be found at ^35^.

The SNP markers were of the codominant type, and the molecular dataset underwent the quality control process carried out in R^36^, using a Call Rate of 90% and MAF (Minor Allele Frequency) of 5%, reducing the marker dataset by 81.75%, starting with 44,457 markers and keeping 8112 for use in the analysis.

### Statistical models

Three models were employed to predict breeding values in the context of genetic improvement: BLUP and two genomic models (GBLUP). The first model considered only the additive effects (GBLUP-A), while the second considered the additive-dominant effects (GBLUP-AD).

*Traditional BLUP* the prediction of breeding values using the BLUP model was performed in R^36^ using the SOMMER package version 3.4. The mixed linear model used was:

$$y=X\mu +Za+\varepsilon $$

where $y$ is the phenotype vector; $X$ is the fixed effect incidence matrix (year); $\mu$ is the fixed effects vector (mean and year); *Z* is the random effects incidence matrix considered as genotype, and *a* is the vector of additive genetic effects considered random with $a \sim N (0, A{\sigma }_{a}^{2})$, where $A$ is the kinship matrix via pedigree. In this case, the relationship is completely unknown; hence $A$ is an identity matrix $I$; ${\sigma }_{a}^{2}$ is the additive genetic variance, and $\varepsilon$ is the residual vector assumed to be random $\varepsilon \sim N \left(0,I{\sigma }_{e}^{2}\right),$ where ${\sigma }_{e}^{2}$ is the residual variance.

*Genomic BLUP (GBLUP)* the GBLUP model is similar to BLUP, except for the assumed kinship matrix. So GBLUP replaces the A matrix with the genomic kinship G matrix derived from the marker information. The genomic breeding values (GEBVs) are predicted using the model below:

$$y=X\mu +Zg+\varepsilon $$

where *g* is the vector of additive genetic effects considered random with $g \sim N (0, G{\sigma }_{g}^{2})$, where $G$ is the additive genomic kinship matrix, and ${\sigma }_{g}^{2}$ is the additive genetic variance. The genomic kinship matrix (G) used was proposed by VanRaden^18^ as follows:

$$ G = \frac{{WW^{\prime } }}{{\mathop \sum \nolimits_{j = 1}^{n} 2p_{j} \left( {1 - p_{j} } \right)}} $$

where *W* is the centered marker matrix as presented by Vitezica et al. (2013), ${W}_{ij}=\left\{2-{2p}_{j},1-{2p}_{j},{-2p}_{j}\right\}$, where ${W}_{ij}$ is the element of the ith row and jth column of the marker matrix, and ${p}_{j}$ is the allele frequency of the marked jth.

*BLUP genomic dominant additive (GBLUP-AD)*

$$y=X\upmu +{Z}_{1}g+{Z}_{2}d+\varepsilon $$

where ${Z}_{1}$ and ${Z}_{2}$ are the incidence matrices of the additive and dominant genetic effects, respectively, considered random; $d$ is the random-effect vector of dominance, where $d\sim N(0, {D\sigma }_{d}^{2})$, *D* is the genomic relationship matrix for dominance effects generated from marker information, and ${\sigma }_{d}^{2}$ is the genetic variance of dominance.

*Dominance genomic kinship matrices (D)* the D matrix was obtained as follows^37–39^:

$$ D = \frac{{SS^{\prime } }}{{{\text{4}}\sum\limits_{{{\text{j = 1}}}}^{{\text{n}}} {\left( {{\text{p}}_{{\text{j}}} \left( {{\text{1 - p}}_{{\text{j}}} } \right)} \right)^{{\text{2}}} } }} $$

where ${S}_{ij}=\left\{-2{\left(1-{p}_{j}\right)}^{2},2{p}_{j}\left(1-{p}_{j}\right),-2{p}_{j}^{2}\right\}$, with ${S}_{ij}$ as the elements of the *it*h row and *j*th column of the *S* matrix, and ${p}_{j}$ as the allele frequency of the *j*th marker.

### Accuracy of genetic values and adjustment of models

The methodologies were compared based on the theoretical accuracy of breeding values (r), variance of the prediction error (PEV), maximum likelihood (LL), Akaike information criterion (AIC), and Bayesian information criterion (BIC).

The r estimates were estimated using the following expression:

$$r=\sqrt{1-\frac{PEV}{{\sigma }_{g}^{2}}}$$

where *PEV* is the variance of the prediction error obtained through the diagonal elements of the mixed model matrix^40^, and ${\sigma }_{g}^{2}$ is the genetic variance.

AIC^41^ was calculated as follows:

$$AIC=2k-2log(L)$$

where *log(L)* is the logarithm of the residual likelihood function (LL), and *k* is the number of estimated parameters.

BIC^42^ can be written as:

$$BIC=-2\mathrm{log}\left(L\right)+k*\mathrm{log}(v)$$

where $v$ is the number of residual degrees of freedom.

### Cross-validation and predictive ability

Predictive ability was estimated by the correlation between the predicted breeding values of each BLUP, GBLUP-A, and GBLUP-AD model ($\widehat{a}$, $\widehat{g}$ e $\widehat{u}=\widehat{g}+\widehat{d}$, respectively) and the phenotypic mean of the evaluated years of each genotype (y). To this end, we used the cross-validation procedure, subdividing the population into five folds. In total, 275 genotypes were used, 220 of which were destined to constitute the training population and 55 to constitute the validation population. The average predictive ability (${r}_{y,\widehat{u}}$) and the standard error were calculated with the results obtained.

### Components of variance and genetic parameters

The variance components were obtained via the REML method. The genetic parameters were estimated by:

$$ H^{2} = \frac{{\sigma_{a}^{2} + \sigma_{d}^{2} }}{{\sigma_{a}^{2} + \sigma_{d}^{2} + \sigma_{e}^{2} }} $$

$$ h_{a}^{2} = \frac{{\sigma_{a}^{2} }}{{\sigma_{a}^{2} + \sigma_{d}^{2} + \sigma_{e}^{2} }} $$

where $H^{2}$ is heritability in the broad sense; ${h}_{a}^{2}$ is heritability in the narrow sense; ${\sigma }_{a}^{2}$ additive genetic variance; ${\sigma }_{d}^{2}$ is the variance related to the dominance deviation, and ${\sigma }_{e}^{2}$ is residual variance.

To verify the influence of insertion of dominance in the model, the relationship was established between ${\sigma }_{a}^{2}$ of the GBLUP-AD model, which for the discussion of this relationship, we will call ${\sigma }_{a(d)}^{2}$, and the ${\sigma }_{a}^{2}$ of the model GBLUP-A.

$${RL}_{aa}=\frac{{\sigma }_{a(d)}^{2}}{{\sigma }_{a}^{2}}$$

Furthermore, to verify the magnitude of the dominance effect as a function of the total variance, a relationship was established between ${\sigma }_{d}^{2}$ and ${\sigma }_{f}^{2}$ of the GBLUP-AD model:

$$ d^{2}  = \frac{{\sigma _{d}^{2} }}{{\sigma _{a}^{2}  + \sigma _{d}^{2}  + \sigma _{e}^{2} }} .$$

### Statement of research involving plants

The experimental research and field studies on plants—Our studied population complies with pertinent legislation. The plant samples were collected in a commercially managed juçaizeiro plantation in the municipality of Rio Novo do Sul in the state of Espírito Santo—Brazil, originating from the process of natural recomposition and seed launch in a pasture area. We had the owners' authorization for all data collected and received support from the companies Açai Juçara and Bonaloti for the research development. We are so grateful to Pedro and Vicente Bortoloti and their families, the owners of managed area. We also confirm that all methods were carried out in accordance with relevant guidelines.

## Results

### Summary of phenotypic data

For all traits, we observed that the sample values evaluated in the genotypes tend to be influenced by the year of evaluation (Table 1). We also found that NBP is the trait that presents the most variable behavior depending on the year. On the other hand, the morphometric traits of fruits and seeds present the most stable phenotypic response among the different evaluated crops (Table 1).

**Table 1:** Summary statistics (mean and standard deviation) of the phenotypic data in the different years of evaluation, adjusted to the model as a fixed effect, for the twelve traits evaluated, as follows: Number of Bunches per Plant (NBP); Fruit Weight per Bunch (kg) (FWB); Rachis Length (cm) (RL); Number of Rachillas per Bunch (NRB); Weight of 25 Fruits (g) (WF); Weight of 25 Seeds (g) (WS); Amount of Pulp in 25 Fruits (g) (AP); Pulp Yield (%) (PY); Equatorial Diameter of Fruits (mm) (EDF); Longitudinal Diameter of Fruits (mm) (LDF); Equatorial Diameter of Seeds (mm) (EDS); Longitudinal Diameter of Seeds (mm) (LDS).

Traits	2018	2019	2020	2021
NBP	2.63 (± 1.54)	3.28 (± 1.36)	3.69 (± 1.03)	2.18 (± 1.36)
FWB	3.05 (± 1.57)	3.45 (± 1.49)	–	3.68 (± 1.87)
RL	60.6 (± 14.35)	62.72 (± 14.15)	–	59.31 (± 10.39)
NRB	107.16 (± 22.04)	114.22 (± 23.46)	–	114.52 (± 21.03)
WF	41.04 (± 8.35)	38.94 (± 7.39)	–	38.15 (± 7.1)
WS	28.6 (± 6.34)	25.04 (± 5.56)	–	26.82 (± 5.75)
AP	12.57 (± 3.59)	13.93 (± 3.07)	–	11.31 (± 2.88)
PY	30.55 (± 6.38)	35.95 (± 5.36)	–	29.78 (± 6.07)
EDF	14.01 (± 1.12)	13.24 (± 1.08)	–	13.31 (± 1.25)
EDS	12.84 (± 1.15)	11.48 (± 1.12)	–	11.73 (± 0.95)
LDF	14.66 (± 1.44)	13.28 (± 1.03)	–	13.58 (± 1.2)
LDS	12.9 (± 1.51)	11.19 (± 0.92)	–	11.12 (± 0.89)

### Estimates of variance components and genetic parameters

Estimates of variance components and genetic parameters for each model are presented in Table 2. Except for the LDS trait, the ${\sigma }_{a}^{2}$ estimates are lower when estimated by the GBLUP-AD model, while the ${\sigma }_{e}^{2}$ estimates indicate small differences between the models. Consequently, the *h*^2^ estimated by the BLUP and GBLUP-A models are generally higher than those estimated by the GBLUP-AD model, which includes both additive and dominance variance effects. The reductions in the ${h}^{2}$ estimates ranged from 0 (LDS) to 0.32 (FWB) between the GBLUP-A and GBLUP-AD models and from 0.03 (LDS) to 0.34 (FWB) between the BLUP and GBLUP-AD models (Table 2).

**Table 2:** Estimates of variance components and genetic parameters estimated by the BLUP, GBLUP-A, and GBLUP-AD models, using information from genotyped trees for the traits number of bunches per plant (NBP); fruit weight per bunch (kg) (FWB); rachis length (cm) (RL); number of rachillas per bunch (NRB); weight of 25 fruits (g) (WF); weight of 25 seeds (g) (WS); amount of pulp in 25 fruits (g) (AP); pulp yield (%) (PY); equatorial diameter of fruits (mm) (EDF); longitudinal diameter of fruits (mm) (LDF); equatorial diameter of seeds (mm) (EDS); longitudinal diameter of seeds (mm) (LDS).

Traits	Model	${\sigma }_{a}^{2}$	${\sigma }_{d}^{2}$	${\sigma }_{e}^{2}$	${h}^{2}$	${H}^{2}$
NBP	BLUPs	0.65	–	1.12	0.37	–
	GBLUP-A	0.64	–	1.16	0.35	–
	GBLUP-AD	0.19	0.33	1.13	0.12	0.31
FWB	BLUP	1.3	–	1.27	0.51	–
	GBLUP-A	1.24	–	1.31	0.49	–
	GBLUP-AD	0.39	0.62	1.27	0.17	0.44
RL	BLUP	59.61	–	126.49	0.32	–
	GBLUP-A	50.39	–	129.3	0.28	–
	GBLUP-AD	35.74	13.7	126.18	0.20	0.28
NRB	BLUP	357.05	–	151.17	0.70	–
	GBLUP-A	357.7	–	153.88	0.70	–
	GBLUP-AD	248.48	72.26	152.11	0.53	0.68
WF	BLUP	54.65	–	9.17	0.86	–
	GBLUP-A	55.45	–	9.22	0.86	–
	GBLUP-AD	36.32	11.42	9.20	0.64	0.84
WF	BLUP	30.06	–	6.59	0.82	–
	GBLUP-A	29.00	–	6.61	0.81	–
	GBLUP-AD	23.17	3.55	6.61	0.70	0.80
AP	BLUP	7.07	–	3.97	0.64	–
	GBLUP-A	6.8	–	4.00	0.63	–
	GBLUP-AD	4.08	1.67	3.99	0.42	0.59
PY	BLUP	17.36	–	17.85	0.49	–
	GBLUP-A	15.26	–	17.87	0.46	–
	GBLUP-AD	13.13	1.35	17.86	0.41	0.45
EDF	BLUP	0.91	–	0.47	0.66	–
	GBLUP-A	0.92	–	0.48	0.66	–
	GBLUP-AD	0.63	0.18	0.48	0.49	0.63
LDF	BLUP	0.82	–	0.59	0.58	–
	GBLUP-A	0.81	–	0.6	0.58	–
	GBLUP-AD	0.61	0.13	0.6	0.46	0.55
EDS	BLUP	0.88	–	0.41	0.68	–
	GBLUP-A	0.88	–	0.41	0.68	–
	GBLUP-AD	0.68	0.12	0.41	0.56	0.66
LDS	BLUP	0.7	–	0.45	0.61	–
	GBLUP-A	0.63	–	0.45	0.58	–
	GBLUP-AD	0.63	0	0.45	0.58	0.58

${\sigma }_{a}^{2}$: additive variance; ${\sigma }_{d}^{2}$: dominance variance; ${\sigma }_{e}^{2}$: residual variance; ${H}^{2}:$ heritability in the broad sense; ${h}^{2}$: heritability in the narrow sense.

Comparing the h^2^ estimates between the BLUP and GBLUP-A models, the greatest difference was observed for the RL characteristic, which was 12.5% smaller when using the genomic model of additive effect (GBLUP-A). When the dominance effect was added to the genomic model (GBLUP-AD), the differences between the h^2^ and the BLUP model intensified, reducing NBP up to 67.57%. In general, the group of traits related to fruit production in the field (NBP, FWB, and RL), except for NBP, showed the greatest changes when considering the dominance effect on the structure of the model.

Overall, H^2^ demonstrated low (RL), medium (NBP, NBP, AP, PY, LDF, and LDS), to high (NRB, WF, WS, EDF, and EDS) heritability. We observed the similarity between the broad-sense heritability (H^2^) estimates by the GBLUP-AD and the narrow-sense heritability (h^2^) obtained by the BLUP and GBLUP-A methods.

Table 3 presents the relationships due to the dominance effect for all traits studied. Except for LDS, which does not have this effect, all other traits have at least a part of their total variance explained by dominance. In this sense, the d^2^ values ranged from 0.04 (PY) to 0.27 (FWB) when excluding the LDS traits. The lower values of ${RL}_{aa}$ and higher values of d^2^ for NBP and FWB indicate the strong influence of dominance for these traits; we noticed that the dominance effect surpassed the additive effect since ${RL}_{aa}$ had values below 0.50 (Table 3).

**Table 3:** Relationships of the dominance effect on the estimation of variance components and genetic parameters between the GBLUP-A and GBLUP-AD models, using information from genotyped trees for the traits number of bunches per plant (NBP); fruit weight per bunch (kg) (FWB); rachis length (cm) (RL); number of rachillas per bunch (NRB); weight of 25 fruits (g) (WF); weight of 25 seeds (g) (WS); amount of pulp in 25 fruits (g) (AP); pulp yield (%) (PY); equatorial diameter of fruits (mm) (EDF); longitudinal diameter of fruits (mm) (LDF); equatorial diameter of seeds (mm) (EDS); longitudinal diameter of seeds (mm) (LDS).

Par	Traits											
	NBP	FWB	RL	NRB	WF	WS	AP	PY	EDF	LDF	EDS	LDS
${RL}_{aa}$	0.30	0.32	0.71	0.69	0.65	0.80	0.60	0.86	0.69	0.75	0.77	1.00
${d}^{2}$	0.20	0.27	0.08	0.15	0.20	0.11	0.17	0.04	0.14	0.10	0.10	0.00

Par.: Parameters; ${RL}_{aa}$: ratio between ${\sigma }_{a}^{2}$ of the GBLUP-AD model and ${\sigma }_{a}^{2}$ of the GBLUP-A model; ${d}^{2}$: relationship between ${\sigma }_{d}^{2}$ and ${\sigma }_{f}^{2}$ of the GBLUP-AD model.

### Adjustment of models and predictive capacity

Table 4 shows the estimates of PEV, r, LL, AIC, and BIC. Based on these results, genomic models GBLUP-A and GBLUP-AD provided a better fit with more accurate breeding value predictions than the conventional BLUP. This is because genomic models generally show lower estimates of PEV, close or higher estimates of theoretical accuracy, and lower values of AIC and BIC compared to conventional BLUP.

**Table 4:** Goodness-of-fit and prediction parameters for genetic values evaluated for the BLUP, GBLUP-A, and GBLUP-AD models using information from genotyped trees for the following traits: number of bunches per plant (NBP); fruit weight per bunch (kg) (FWB); rachis length (cm) (RL); number of rachillas per bunch (NRB); weight of 25 fruits (g) (WF); weight of 25 seeds (g) (WS); amount of pulp in 25 fruits (g) (AP); pulp yield (%) (PY); equatorial and longitudinal diameter of fruits and seeds (mm) (EDF, LDF, EDS, and LDS; respectively).

Traits	Models	PEV	$r$	LL	AIC	BIC
NBP	BLUP	0.21	0.82	− 345.05	698.1	717.82
	GBLUP-A	0.19	0.83	− 348.71	705.42	725.14
	GBLUP-AD	0.15	0.84	− 340.83	689.67	709.39
FWB	BLUP	0.39	0.83	− 257.97	521.93	535.38
	GBLUP-A	0.36	0.84	− 254.04	514.08	527.52
	GBLUP-AD	0.30	0.84	− 249.55	505.1	518.55
RL	BLUP	28.67	0.72	− 299.72	605.44	618.89
	GBLUP-A	24.28	0.72	− 291.64	589.28	602.73
	GBLUP-AD	22.64	0.74	− 290.89	587.78	601.23
NRB	BLUP	57.62	0.92	− 181.88	369.77	383.21
	GBLUP-A	53.98	0.92	− 171.95	349.9	363.35
	GBLUP-AD	91.02	0.85	− 170.75	347.5	360.94
WF	BLUP	1.25	0.99	599.6	− 1193.19	− 1175.66
	GBLUP-A	1.05	0.99	613.54	− 1221.08	− 1203.55
	GBLUP-AD	10.36	0.88	616.67	− 1227.34	− 1209.81
WS	BLUP	0.87	0.99	422.77	− 839.54	− 822.05
	GBLUP-A	0.75	0.99	443.65	− 881.3	− 863.81
	GBLUP-AD	4.16	0.92	444.53	− 883.06	− 865.57
AP	BLUP	0.47	0.97	− 302.46	610.93	628.41
	GBLUP-A	0.43	0.97	− 287.92	581.84	599.32
	GBLUP-AD	1.5	0.86	− 283.82	573.64	591.12
PY	BLUP	1.94	0.94	− 467.05	940.1	957.58
	GBLUP-A	1.76	0.94	− 437.3	880.6	898.08
	GBLUP-AD	2.81	0.90	− 436.97	879.94	897.42
EDF	BLUP	0.06	0.97	− 285.78	577.56	595.05
	GBLUP-A	0.06	0.97	− 273.44	552.88	570.37
	GBLUP-AD	0.19	0.88	− 271.87	549.75	567.24
LDF	BLUP	0.07	0.95	− 262.42	530.85	548.34
	GBLUP-A	0.07	0.96	− 247.17	500.34	517.84
	GBLUP-AD	0.16	0.88	− 245.88	497.77	515.26
EDS	BLUP	0.06	0.97	− 50.26	106.52	123.9
	GBLUP-A	0.05	0.97	− 35.53	77.06	94.44
	GBLUP-AD	0.16	0.90	− 34.56	75.11	92.49
LDS	BLUP	0.06	0.96	27.91	− 49.82	− 32.43
	GBLUP-A	0.05	0.96	57.36	− 108.72	− 91.33
	GBLUP-AD	0.05	0.96	57.36	− 108.72	− 91.33

The Maximum Likelihood Ratio Test (LRT) between genomic models indicated a significant dominance effect for the traits FWB, NBP, WF, and AP, as seen in Table 3 by higher values of d^2^ (0.27, 0.20, 0.20, and 0.17, respectively) and lower values of ${RL}_{aa}$ (0.32, 0.30, 0.65, and 0.60, respectively). For the other variables, the GBLUP-AD and GBLUP-A models showed no statistical differences by the LRT. However, it is worth mentioning that r had higher values for the RL trait, and AIC and BIC presented lower values for the model that considers the dominance effect, demonstrating higher performance. For NR, even with better-fit quality indicated by AIC and BIC, r and PEV for GBLUP-AD were lower.

**Figure 2:**
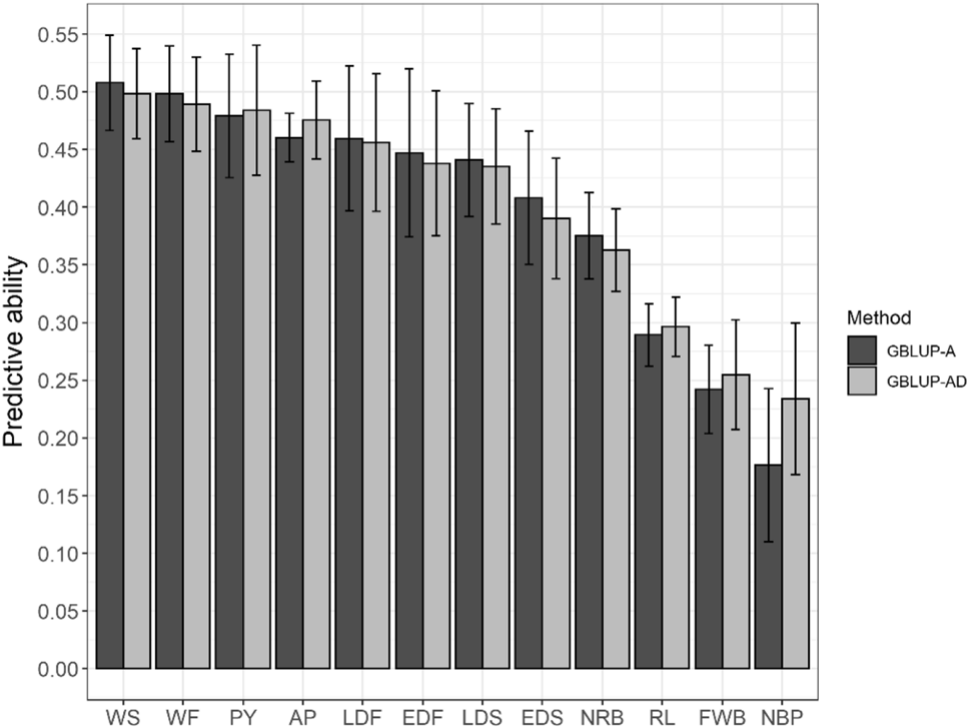
Standard error of the mean and mean predictive ability (${r}_{y,\widehat{u}})$ of the GBLUP-A and GBLUP-AD models for the traits number of bunches per plant (NBP); fruit weight per bunch (kg) (FWB); rachis length (cm) (RL); number of rachillas per bunch (NRB); weight of 25 fruits (g) (WF); weight of 25 seeds (g) (WS); amount of pulp in 25 fruits (g) (AP); pulp yield (%) (PY); equatorial and longitudinal diameter of fruits and seeds (mm) (EDF, LDF, EDS, and LDS; respectively).

The lowest estimates of $r$ were observed for LR: 0.72 (BLUP), 0.72 (GBLUP-A), and 0.74 (GBLU-AD). However, the results obtained for the other traits had a high magnitude, ranging from 0.82 (NBP) to 0.99 (WF and WS). For all traits, the predictive capabilities (${r}_{y,\widehat{u}})$ of BLUP were equal to zero. For the genomic methods, ${r}_{y,\widehat{u}}$ (Fig. 2) ranged from 0.18 (NBP) to 0.51 (WS) for GBLUP-A and from 0.23 (NBP) to 0.50 (WS) for GBLUP-AD.

Under five-fold cross-validation, a slight difference was observed between the genomic models, with the difference more pronounced for NBP. The dominance effect was more pronounced for NBP, reflecting the better performance of the GBLUP-AD model (Table 4). This is expressed by ${r}_{y,\widehat{u}}$, which increased from 0.18 to 0.23 (Fig. 2).

Furthermore, except for NR, the traits related to fruit production in the field (CR, MFC, and NC) presented lower ${r}_{y,\widehat{u}}$, with estimates below 0.30. The morphometric traits of fruits and seeds (MFS, MFF, REND, QP, DLF, DEF, DLS, and DES) had a higher performance for GEBVs, with ${r}_{y,\widehat{u}}$ above 0.35 (Fig. 2).

## Discussion

### Summary of phenotypic data

Assessing the productive behavior of juçaizeiro (Table 1) is of great importance for growers and stakeholders involved in the crop as it enables an understanding of the productive behavior of this plant and facilitates future estimates of output and profit. It is also worth mentioning that the experimental area evaluated received no cultivation treatments, and therefore, the productive aspects have significant potential for increasing yield by implementing management practices that lead to short-term changes. Added to this, for the crop as a whole, there is a high potential that genetic advances can be obtained in the juçaizeiro breeding programs in the coming years, which will contribute to establishing the species in the fruit sector and maximizing economic gains in fruit production.

### Genomic selection and dominance effect in Juçaizeiro breeding

In the present study, BLUP, GBLUP-A, and GBLUP-AD were evaluated to estimate the variance components, genetic parameters, prediction accuracies, and predictive abilities, with the objective of assessing the efficiency of genomic selection and genetic control of several productive traits of the juçaizeiro. This species is in the early stages of improvement, and our study aimed to provide information on its genetic control of several traits of interest to fruit production.

As this crop is only found in forest fragments with small commercial orchards, few improvement studies, which address only fruit, seed, and germination traits, have been carried out with it^43–46^. Additionally, only additive effects are incorporated into the genetic value prediction models, which are unaware of the kinship between individuals. In contrast, we utilized not only morphometric aspects but also data related to fruit yield and incorporated dominance effects into additive models through the knowledge of kinship among individuals. Therefore, this is the first study to include dominance in the genetic control study of juçaizeiro traits.

To better understand the importance of knowledge about the genetic control structure of productive traits in the juçaizeiro, some specific aspects related to species breeding must be presented. The species is currently propagated exclusively by seeds, and breeding programs primarily focus on understanding the additive genetic control for estimating and selecting the best genotypes based solely on these effects^33,45,46^, since the dominance effects will not be transmitted to the selected materials due to the propagation method used.

Although dominance is not considered in seed recommendations for commercial plantations, it plays a crucial role in the species' breeding program. Thus, a deeper understanding of the genetic control structure and the magnitude of these effects under the traits of interest is essential for decision-making by genetics breeders. By quantifying the effects that explain the observed phenotypic variation, breeders can optimize selective gains and use more effective methods.

Moreover, assessing genetic control and the magnitude of the effects of genetic dominance is necessary for making informed decisions about genetically complementary breeding. This understanding enables breeders to identify the most effective crosses to achieve desirable traits^47^, as crosses of the best genotypes based solely on additive effects may not provide the best progenies^48,49^. Thus, knowledge of dominance associated with genetic divergence among individuals becomes essential for promoting the capitalization of the heterotic effect through controlled crosses, seeking better results in the population of the second selection cycle.

In addition to being a crucial aspect for developing offing hybrids, understanding the dominance effect is fundamental to detecting disturbing effects in selecting superior genetic materials^50^. However, in classical breeding programs, estimating these variance components becomes unfeasible due to the requirement to conduct a large number of controlled crosses and experimental areas. Moreover, crop development time can delay the species improvement program, taking at least 10 to 15 years to estimate genetic effects such as dominance, considering that the tested materials originate from controlled crosses. Thus, genomic techniques have proven effective for the juçaizeiro by providing rapid, in-depth information about the genetic control of its traits and improving estimates of additive genetic effects.

In this sense, the genomic methods applied in the present work already surpass the traditional BLUP method since it does not allow estimation of dominance effects due to the lack of pedigree information. Nevertheless, these more sophisticated genomic techniques offer advantages as they generate increased selective accuracy and allow more extensive studies of the genetic control of traits in a more immediate way, while traditional methods would require the performance of controlled crosses (prone to errors and contamination by pollen)^51^, long periods for plant development, and large experimental areas.

To compare the methodologies, the quality measures of AIC and BIC adjustments were used. Through them, we were able to conclude that the inclusion of dominance effects improves model fit for all evaluated traits, as evidenced by the smaller AIC and BIC values (Table 4). Conversely, when using the BLUP model for all evaluated traits, the goodness-of-fit measures were superior to the other models (Table 4). However, including the dominance effects may lead to negative responses in predicting some traits, as shown by the increase in PEV and, consequently, a slight reduction in $r$ (Table 4). Including dominance effects in the model brought more noticeable improvement to the traits FWB, NBP, WF, and AP. Therefore, the ${r}_{y,\widehat{u}}$ values considering dominance were superior to the GBLUP-A models, indicating the importance of including this effect in the additive model for estimating individual GEBVs.

As previously mentioned, the lower $r$ values observed for the traits generally have a high magnitude, and even though the LRT revealed the non-existence of differences among traits between the methods, the use of the dominance effect is justified for understanding genetic control of the traits.

In the present study, the differences observed between the BLUP and GBLU-A models as a function of h^2^ did not have high magnitudes. This same behavior was observed in the morphological traits of pine^52^. However, in contrast to these observations, studies conducted on *Eucalyptus* found more pronounced changes between the estimates^14,15^. Nevertheless, in the cited studies, the drastic changes that can be achieved using marker-based models compared to pedigree information are evident; this was also observed in the present study when the dominance effect was added to the model, which can correct for overestimations in additive genetic variance, as shown by the reduction in the h^2^ estimates (Table 2), agreeing as well with studies on maize^53^ and eucalyptus^54^.

The similarity observed between broad-sense heritability estimates (H^2^) obtained by GBLUP-AD and narrow-sense heritability (h^2^) obtained by GBLUP-A may be associated with the fact that the type of regularization used in the marker matrix for estimating additive effects using the VanRaden (2008) method^18^ makes the additive effects of markers correspond to the allelic substitution effect, whose estimation terms contain both the additive effect of the homozygote and the dominant effect of the heterozygote $\alpha =a+d\left(q-p\right),$ leading us to believe the dominance effect is confounded in this estimate, resulting in overestimations by this latter model. In conclusion, the ${\sigma }_{a}^{2}$ estimated by GBLUP-A is close to the ${\sigma }_{g}^{2}$ estimates obtained by GBLUP-AD, resulting in similar h^2^ and H^2^ estimates between the methods.

In addition, the use of molecular markers shows great relevance for a more in-depth investigation of the genetic control of traits and allows more realistic gains estimates. The observed broad-sense heritability (H^2^), which shows how much of the phenotype variation is explained by the genotype, can be classified as low (RL) (< 0.30), medium (NBP, FWB, AP, PY, and LDS ) (0.30–0.60), and high (NRB, WF, WS, EDF, LDF, and EDS) (> 0.60)^55^. The results shown in Table 2 reveal that traits associated with fruit production (NBP, FWB, RL, and NRB) are more influenced by environmental effects than morphometric variables (WF, WS, AP, PY, EDF, LDF, EDS, and LDS). Among production-related traits, NRB stands out from the others in terms of genetic control, with a H^2^ of 0.68 classified as high.

Adding dominance effects to the predictive models can increase the predictive ability of the genomic model, with the improvement determined by the proportion in which this effect can explain the total variation. This behavior can be observed mainly for the traits FWB (Table 3) and NBP (Table 4), which showed the greatest influence of dominance. Improvement in models incorporating dominance for traits with higher d^2^ can also be observed in simulated data from pine populations^49^. Such improvements are fundamental for the juçaizeiro. Although not propagated clonally, removing the dominance effects from estimates of additive effects can increase selective accuracy and promote greater future gains, highlighting the relevance of genomic techniques in the studied population.

Therefore, the presented results contribute to improving the understanding of the genetic structure that governs part of the phenotypic variation of quantitative traits of productive and industrial interest. Moreover, the importance of including the dominance effect to obtain a more realistic partitioning of genetic variance in its additive and non-additive terms is evident by the reduction of h^2^ for most traits.

### Predictive ability

Predictive ability is a striking measure of comparison among the models tested in this study. Since conventional BLUP does not provide any pedigree information, the genetic values predicted by this method for untested materials correspond to the average; consequently, the predictive ability becomes zero. For the models based on genomic information, estimates of ${r}_{y,\widehat{u}}$ among the traits are distinct and associated with several factors, such as h^2^. This is further evidence of the superiority of genomic methods over the traditional method.

In general, two factors affect the predictive ability of marker-based models, including heritability, which is dependent on the genetic control of the trait, and phenotyping quality, associated with field evaluation processes^56^. We found estimates of ${r}_{y,\widehat{u}}$ with mean values below 0.55; Since this is an initial study applying genomic techniques in natural populations, hypotheses were raised for techniques that require complementary studies to improve the results observed for the experimental condition tested.

In this sense, it is possible that including covariates associated with plant age, like height and circumference, could improve the performance of predictions and covariates associated with the plot conditions where a group of plants is located. Additionally, the trait-assisted method of genomic prediction^57^, with or without covariates, may be an alternative to maximize predictive abilities. This method not only uses molecular marker information but also benefits from the correlated effects among the traits of individuals whose genetic value is being predicted to improve the predictions of a key trait.

In general, the values of ${r}_{y,\widehat{u}}$ obtained using the GBLUP-A and GBLUP-AD methods were similar, with a greater difference observed for NBP (Table 4). Such similarity associated with predictive relationships between models has been observed in the literature^58–60^. In this sense, Azevedo et al. (2015)^60^ concluded that other parameters should be considered for model comparison. The authors determined that estimates of heritability, bias, and the relationship between variation due to dominant and additive effects are more relevant factors for such a comparison. Therefore, in the present study, as the GBLUP-AD model presented the best fitting parameters (AIC and BIC) and demonstrated that GBLUP-A may have confounded its additive term with dominance effect, overestimating h^2^, we conclude that the model that included the dominance term improved the selection of superior genetic materials, with GBLUP-AD superior to GBLUP- A.

## Conclusion

Our findings on model adjustment parameters and predictive capabilities show that genomic methods are superior to conventional BLUP for the juçaizeiro. The superiority of genomic prediction is further confirmed by the fact that it allows a more in-depth study of the genetic control of traits, which is not possible by BLUP due to the complete absence of knowledge of kinship between individuals. To generate such information, this method would require great effort and financial resources, in addition to the time that would easily exceed 15 years of research.

For all traits but LDS, which presented variance due to dominance equal to zero, the models that considered only the additive effects presented estimates of the variance components and genetic parameters greater than the GBLUP-AD model, indicating that including the dominance effect corrects overestimations, returning more real estimates. Thus, genetic gains can be estimated with greater accuracy from the results generated by the GBLUP-AD model.

Among the methods and models tested (BLUP, GBLUP-A, and GBLUP-AD), GBLUP-AD had the best results, demonstrating the importance of estimating the dominance effect for productive traits in juçaizeiro.

## Data Availability

The datasets generated during and/or analyzed during the current study are available from the corresponding author on reasonable request and with permission of W agency.

## References

[1] Bourscheid, K., Siminski, A., Fantini, A. C. & Mac Faden, J. Euterpe edulis. *Espécies Nativ. da flora Bras. valor econômico atual ou potencial plantas para o Futur. Sul. Brasília MMA* 178–183 (2011).

[2] Reitz, R. Palmeiras In: REITZ, R. *Flora Ilus. Catarinense. Itajaí Herbário Barbosa Rodrigues* (1974).

[3] de Maciel LO, de Moura NF, Leonardi A (2019). Cadeia produtiva do açaí juçara na região do litoral norte do Rio Grande do Sul. Rev. Teor. e Evidência Econômica.

[4] de Carvalho LMJ, Esmerino AA, de Carvalho JLV (2022). Jussaí (Euterpe edulis): A review. Food Sci. Technol..

[5] Felzenszwalb I, Marques MRC, Mazzei JL, Aiub CAF (2013). Toxicological evaluation of Euterpe edulis: A potential superfruit to be considered. Food Chem. Toxicol..

[6] de Oliveira Ribeiro L, Mendes MF, de Pereira CSS (2011). Avaliação da composição centesimal, mineral e teor de antocianinas da polpa de juçaí (*Euterpe edulis* Martius). Rev. Eletrônica TECCEN.

[7] da Chaves SSF, Alves RM, dos Dias LAS (2021). Contribution of breeding to agriculture in the Brazilian amazon I. Açaí palm and oil palm. Crop Breed. Appl. Biotechnol..

[8] Pereira AG (2022). Patterns of genetic diversity and structure of a threatened palm species (*Euterpe edulis* Arecaceae) from the Brazilian Atlantic forest. Heredity (Edinb).

[9] Gaiotto FA, Grattapaglia D, Vencovsky R (2003). Genetic structure, mating system, and long-distance gene flow in heart of palm (*Euterpe edulis* Mart.). J. Hered..

[10] Henderson A, Galeano G, Bernal R (1995). Field guide to the palms of the Americas.

[11] Piaskowski J (2018). Genomic heritability estimates in sweet cherry reveal non-additive genetic variance is relevant for industry-prioritized traits. BMC Genet..

[12] Ratcliffe B (2017). Single-step BLUP with varying genotyping effort in open-pollinated Picea glauca. G3 Genes Genomes Genet.

[13] Crossa J (2010). Prediction of genetic values of quantitative traits in plant breeding using pedigree and molecular markers. Genetics.

[14] Thavamanikumar S, Arnold RJ, Luo J, Thumma BR (2020). Genomic studies reveal substantial dominant effects and improved genomic predictions in an open-pollinated breeding population of Eucalyptus pellita. G3 Genes Genomes Genet.

[15] Gamal El-Dien O (2016). Implementation of the realized genomic relationship matrix to open-pollinated white spruce family testing for disentangling additive from nonadditive genetic effects. G3 Genes Genomes Genet.

[16] Tambarussi EV, Pereira FB, da Silva PHM, Lee D, Bush D (2018). Are tree breeders properly predicting genetic gain? A case study involving Corymbia species. Euphytica.

[17] Paludeto JGZ, Grattapaglia D, Estopa RA, Tambarussi EV (2021). Genomic relationship–based genetic parameters and prospects of genomic selection for growth and wood quality traits in Eucalyptus benthamii. Tree Genet. Genomes.

[18] VanRaden PM (2008). Efficient methods to compute genomic predictions. J. Dairy Sci..

[19] Grattapaglia D (2018). Quantitative genetics and genomics converge to accelerate forest tree breeding. Front. Plant Sci..

[20] Pascual L (2020). Genomic analysis of Spanish wheat landraces reveals their variability and potential for breeding. BMC Genom.

[21] Shams F (2019). Application of DArT seq derived SNP tags for comparative genome analysis in fishes; An alternative pipeline using sequence data from a non-traditional model species Macquaria ambigua. PLoS ONE.

[22] Sansaloni C (2020). Diversity analysis of 80,000 wheat accessions reveals consequences and opportunities of selection footprints. Nat. Commun..

[23] Almeida Filho JE (2019). Genomic prediction of additive and non-additive effects using genetic markers and pedigrees. G3 Genes Genomes Genet..

[24] Duenk P, Calus MPL, Wientjes YCJ, Bijma P (2017). Benefits of dominance over additive models for the estimation of average effects in the presence of dominance. G3 Genes Genomes Genet..

[25] Sun C, VanRaden PM, Cole JB, O’Connell JR (2014). Improvement of prediction ability for genomic selection of dairy cattle by including dominance effects. PLoS One.

[26] Denis, M., Bouvet, J.-M. Genomic selection in tree breeding: testing accuracy of prediction models including dominance effect. In *BMC Proceedings* vol. 5 1–2 (Springer, 2011).

[27] R Core Team. A language and environment for statistical computing. (2022).

[28] David K, Hadley W (2013). ggmap: Spatial visualization with ggplot2. R J..

[29] Pereira, R. H. M. & Goncalves, C. N. geobr: Download official spatial data sets of Brazil. (2023).

[30] Hadley Wickham. ggplot2: Elegant graphics for data analysis. (2016) doi:978–3–319–24277–4.

[31] de Marçal TS (2016). Repeatability of biometric characteristics of Juçara palm fruit. Biosci. J..

[32] Doyle JJ (1990). Isolation of plant DNA from fresh tissue. Focus (Madison)..

[33] Carvalho MS (2020). Genetic diversity and population structure of *Euterpe edulis* by REML/BLUP analysis of fruit morphology and microsatellite markers. Crop Breed. Appl. Biotechnol..

[34] Kilian, A. *et al.* Diversity arrays technology: a generic genome profiling technology on open platforms. In *Data production and analysis in population genomics* 67–89 (Springer, 2012). 10.1007/978-1-61779-870-2_5 22665276

[35] Canal GB (2023). Single and multi-trait genomic prediction for agronomic traits in Euterpe edulis. PLoS One.

[36] RDC, T. A Language and environment for statistical computing. *Vienna, Austria: R Foundation for Statistical Computing* (2010).

[37] Vitezica ZG, Varona L, Legarra A (2013). On the additive and dominant variance and covariance of individuals within the genomic selection scope. Genetics.

[38] Aliloo H (2017). Including nonadditive genetic effects in mating programs to maximize dairy farm profitability. J. Dairy Sci..

[39] Zhang H, Yin L, Wang M, Yuan X, Liu X (2019). Factors affecting the accuracy of genomic selection for agricultural economic traits in maize, cattle, and pig populations. Front. Genet..

[40] Gilmour AR, Thompson R, Cullis BR (1995). Average information REML: An efficient algorithm for variance parameter estimation in linear mixed models. Biometrics.

[41] Akaike H (1974). A new look at the statistical model identification. IEEE Trans. Automat. Contr..

[42] Schwarz G (1978). Estimating the dimension of a model. Ann. Stat..

[43] Carvalho HF (2020). The effect of bienniality on genomic prediction of yield in arabica coffee. Euphytica.

[44] Marçal TDES, Ferreira A, Oliveira WBDOSS, Guilhen JHS, Ferreira MFDAS (2015). Correlações genéticas e análise de trilha para caracteres de fruto da palmeira juçara. Rev. Bras. Frutic..

[45] de Marcal TS (2020). Genetic diversity of Euterpe edulis martius based on fruit traits. Biosci. J..

[46] Soler-Guilhen JH (2020). Euterpe edulis seed germination parameters and genotype selection. Acta Sci. Agron..

[47] Toro MA, Varona L (2010). A note on mate allocation for dominance handling in genomic selection. Genet. Sel. Evol..

[48] Gerhardt IFS (2019). Genetic effects on the efficiency and responsiveness to phosphorus use in popcorn as estimated by diallel analysis. PLoS ONE.

[49] de Almeida Filho JE (2016). The contribution of dominance to phenotype prediction in a pine breeding and simulated population. Heredity (Edinb).

[50] Cruz, C. D. *Princípios de Genética quantitativa*. (2012).

[51] Tan B, Grattapaglia D, Wu HX, Ingvarsson PK (2018). Genomic relationships reveal significant dominance effects for growth in hybrid Eucalyptus. Plant Sci..

[52] Muñoz PR (2014). Unraveling additive from nonadditive effects using genomic relationship matrices. Genetics.

[53] Dias KODG (2018). Improving accuracies of genomic predictions for drought tolerance in maize by joint modeling of additive and dominance effects in multi-environment trials. Heredity (Edinb)..

[54] Bouvet J-M, Makouanzi G, Cros D, Vigneron PH (2016). Modeling additive and non-additive effects in a hybrid population using genome-wide genotyping: prediction accuracy implications. Heredity (Edinb)..

[55] Johnson HW, Robinson HF, Comstock RE (1955). Estimates of genetic and environmental variability in soybeans 1. Agron. J..

[56] Combs E, Bernardo R (2013). Accuracy of genomewide selection for different traits with constant population size, heritability, and number of markers. Plant Genome.

[57] Fernandes SB, Dias KOG, Ferreira DF, Brown PJ (2018). Efficiency of multi-trait, indirect, and trait-assisted genomic selection for improvement of biomass sorghum. Theor. Appl. Genet..

[58] Gianola D (2013). Priors in whole-genome regression: the Bayesian alphabet returns. Genetics.

[59] de Los Campos G, Hickey JM, Pong-Wong R, Daetwyler HD, Calus MPL (2013). Whole-genome regression and prediction methods applied to plant and animal breeding. Genetics.

[60] Azevedo CF (2015). Ridge, lasso and bayesian additive-dominance genomic models. BMC Genet..

